# 80 Years of vision: preventing blindness from retinopathy of prematurity

**DOI:** 10.1038/s41372-021-01015-8

**Published:** 2021-03-05

**Authors:** Edward H. Wood, Emmanuel Y. Chang, Kinley Beck, Brandon R. Hadfield, Amy R. Quinn, Clio Armitage Harper

**Affiliations:** 1 Byers Eye Institute, Horngren Family Vitreoretinal Center, Department of Ophthalmology, Stanford University School of Medicine, Palo Alto, CA USA; 2 Stanford Cardiovascular Institute, Stanford University School of Medicine, Palo Alto, CA USA; 3 Retina and Vitreous of Texas, Houston, TX USA; 4 Eyesight Ophthalmic Services, Portsmouth, NH USA; 5 University of Texas Health Science Center San Antonio, Department of Pediatrics, Division of Neonatology, San Antonio, TX USA; 6 Austin Retina Associates, Austin, TX USA; 7 University of Texas Health Science Center San Antonio, Department of Ophthalmology, San Antonio, TX USA; 8 University of Texas at Austin, Department of Ophthalmology, Austin, TX USA

**Keywords:** Diseases, Pathogenesis

## Abstract

Retinopathy of prematurity (ROP) is one of the leading yet preventable causes of childhood blindness worldwide. The purpose of this review is to provide a practical template for observational and treatment methods in order to reduce the overall incidence of any ROP and to improve both short-term and long-term outcomes once Type 1 ROP (treatable ROP) develops.

## Introduction

Retinopathy of Prematurity (ROP) is one of the leading, yet largely preventable causes of childhood blindness in the United States and worldwide. Globally, at least 50,000 children are blind as a result of ROP and in the United States, even with many advances in ROP treatment, approximately 600 premature infants become legally blind each year [1, 2]. The purpose of this editorial is to provide a practical, algorithmic template for observational and treatment methods in order to reduce the overall incidence of any ROP and to improve both short-term and long-term outcomes once Type 1 ROP (treatable ROP) develops.

Recent concepts will be reviewed, including preventative strategies that may be employed in the neonatal intensive care unit (NICU), the role of laser in the treatment of Type 1 ROP and in order to complete the treatment cycle after anti-vascular endothelial growth factor (anti-VEGF) treatment, intravitreal anti-VEGF drugs currently in use for the treatment of Type 1 ROP, the SAFER method for dependable and standardized intravitreal injection technique, a brief discussion of the evidence of neurodevelopmental changes following anti-VEGF treatment, the role of fluorescein angiography (FA) evaluation following anti-VEGF injection and in cases with persistent mild ROP, the necessity of careful and consistent follow-up after the treatment with intravitreal anti-VEGF drugs as well as laser, and finally genetic variants that may predispose to more persistent or severe ROP.

## The role of neonatology and target oxygen saturation

Since retinopathy of prematurity (ROP) was first noted in 1942 [3], much has been done in an attempt to prevent and treat ROP. Known risk factors of ROP include earlier gestational age and lower birth weight. Since as early as the 1950s, controlling oxygen therapy has been the first-line preventative measure in ROP [4–6]. This control is maintained by adjusting pulse oximetry saturation targets which are used to titrate the fraction of inspired oxygen (FiO2) in order to achieve appropriate oxygenation while avoiding detrimental systemic effects of oxidant stress (most notably bronchopulmonary dysplasia and ROP). The “Supplemental Therapeutic Oxygen for Prethreshold Retinopathy of Prematurity” (STOP-ROP) study randomized babies with “prethreshold ROP” to a pulse oximetry target of 89–94% vs. 96–99%. STOP-ROP found that the higher oxygen target group had minimal improvement in ROP progression, but an increase in pulmonary adverse events [7]. The “Benefits of Oxygen Saturation Targeting” (BOOST II) study randomized infants born before 28 weeks’ gestation to a pulse oximetry target of 85–89% vs. 91–95%, and found no difference in growth or neurodevelopmental outcomes in either group but did note increased mortality and dependence on supplemental oxygen at 36 weeks in the lower oxygen target group, ultimately prompting early study termination [8].

It was clear that target oxygen saturation played a role in ROP, and further studies found relative success in matching oxygen saturation targets with the two phases of ROP that occur in the development of the neonatal retina [7, 9–13].

In Phase 1, requisite oxygen supplementation leads to relative retinal hyperoxygenation (supply > demand) caused by diffusion of oxygen from the choriocapillaris, a highly vascularized layer of unfenestrated blood vessels beneath the retina, to peripheral areas of undeveloped retina. Relative retinal hyperoxygenation results in retinal vascular growth attenuation and vaso-obliteration with subsequent areas of peripheral retinal avascularity [14]. In Phase 2, the retina matures resulting in relative retinal hypo-oxygenation (demand > supply), leading to an acute overexpression of cytokines (including VEGF) from the aforementioned avascular areas ultimately culminating in pathologic angiogenesis. This theory prompted multiple studies utilizing a biphasic approach to oxygenation in the treatment and prevention of ROP, with a lower target SpO2 in infants <33 weeks of age and a higher target SpO2 in infants >34 weeks of age [7, 9–13].

In 2019 Shukla et al [6]. published a retrospective cohort study comparing biphasic versus static standards 41 months prior to and 42 months after a change from biphasic to static support standards at a level III neonatal intensive care unit. The pre-support group underwent biphasic protocol target saturations of 85 to 92% at younger than 34 weeks corrected gestational age (CGA) and greater than 95% at 34 weeks CGA or older. The post-support group underwent a constant 91 to 95% target. 562 infants were included in ophthalmic analysis. The incidence of Type 1 ROP was 2% in the biphasic (presupport) group versus 6% in the static oxygen (post-support) group. In addition, the data demonstrated that relative hypoxia in early gestation and increase in oxygen saturation later in gestation is associated with reduced ROP but not increased mortality risk.

In our clinical practice, we consider a modified approach to the application of the oxygen saturation target studies that we have termed “Triphasic Oxygen for Prevention of ROP” (TOP-ROP). It involves a multi-disciplinary approach in which there is close coordination between the screening ophthalmologist, the neonatologist, and the nurses involved in direct care. The first phase of oxygen saturation targets is set by the NICU and unchanged if the child does not become at-risk for Type 1 ROP. If the child is beyond 33 weeks PMA and exhibits worsening disease that appears to be approaching the requirement for treatment (i.e increasing vascular tortuosity and/or Stage 2 ROP), the second phase of increasing oxygen saturation targets to 95–99% is considered. This is done in order to reduce the progression mild ROP to Type 1 ROP, and is discussed with the neonatologist to ensure that increasing saturation targets would not be detrimental to the developing pulmonary status (especially in severe bronchopulmonary dysplasia cases). If a reduction in mild ROP is noted, then the oxygen is gradually weaned. If the infant progresses to Type 1 ROP and is treated with anti-VEGF therapy, the third phase is discussed which is reducing oxygen saturation targets in conjunction with the neonatologist. This is done in order to prevent oxygen-induced retinopathy that may play a role in disease re-activation after anti-VEGF therapy, especially in older infants that remain on medium to long-term oxygen in the outpatient setting.

The “Weight, insulin-like growth factor 1, neonatal retinopathy of prematurity” (WINROP) study sought to develop a calculator to aid in determining which infants needed to be monitored and screened for ROP. While the study found that nutrition and growth were related to a decrease in ROP rates [15], the study was limited to a homogenous patient population and may not be applicable to diverse clinical practices. In line with maximizing nutrition, other modalities utilized to decrease ROP rates include avoiding hyperglycemia [16], utilizing appropriate Omega 3 fatty acid supplementation [17], decreasing when possible total parenteral nutrition (TPN) [18], and minimizing anemia [19].

In general, neonatologists and ophthalmologists should be aware of patients that have been treated with intravitreal anti-VEGF. There is still a lack of data for the best course of action, but the TOP-ROP oxygen saturation target regimen has the potential to become a helpful tool in the prevention and treatment of ROP. Further prospective, multicenter trials are needed to confirm both the effect on the prevention of Type 1 ROP and systemic outcomes.

## When to treat, the ideal treatment, and the current treatments

If ROP is classified as Type 1 [20], treatment should be planned and performed within 48–72 h per the Early Treatment of Retinopathy of Prematurity (ETROP) cooperative group [20], with more severe cases treated sooner. The simplified concept underlying the treatment of ROP is that the avascular retina (the ‘messenger’) produces an overabundance of VEGF (the ‘message’) that results in the growth of abnormal blood vessels (neovascularization) along the retinal surface and into the vitreous cavity. The ideal therapy for ROP (which does not yet exist) would simultaneously suppress neovascularization, reliably enable complete physiologic retinal vascularization, support healthy neural development of the neonatal retina, and exhibit no local or systemic side effects. Our current treatment options can quiet the messenger (ablate the peripheral retina with laser photocoagulation), temporize the message (intravitreal anti-VEGF injections), or reduce anatomical changes caused by fibrosis that accompanies neovascularization (vitreoretinal surgery). We will discuss below high yield considerations for each therapeutic modality available for Type 1 ROP.

## Treatment: laser photocoagulation

Originally, cryotherapy was utilized to destroy the peripheral retina by freezing the avascular retina through the external scleral wall [21]. Although effective and revolutionary at the time, the side effect profile is high relative to modern techniques and it is rarely used today. The mainstay treatment of ROP for many years is ablative indirect diode laser photocoagulation to the avascular peripheral retina. In the landmark ETROP study, which began enrolling patients in 2000, laser ablation was shown to reduce unfavorable structural outcomes from 15.6 to 9.0 percent at 9 months [20].

When the decision is made to use laser therapy either to treat Type 1 ROP or following anti-VEGF therapy, the authors prefer to use the Iridex 810 nanometer (nm) laser connected to a laser indirect ophthalmoscope (LIO) and a 28 diopter (D) condensing lens. 810 nanometer laser is preferred because of its decreased absorption by the tunica vasculosa lentis, and therefore more efficient uptake to the retina and less cataract formation [22]. The authors begin by performing a fluorescein angiogram (FA) using 7.7 mg/kg of intravenous fluorescein, followed by applying grayish burns anterior to the ridge and posterior to the ora serrata for 360 degrees in a “near-confluent” pattern (½ spot width separation) aside from temporally over the ciliary arteries where 1 spot width separation is used. Attention is drawn to the temporal notch and the presence of flat neovascularization which, once regressed, may unveil subsequent avascular retina requiring sequential laser [23].

Detailed informed consent is critical when administering any treatment for ROP. When treating with laser, it is important to discuss the relative certainty of decreased peripheral vision, decreased night vision, and myopia. Myopia is a feature of ROP (and prematurity itself), but the degree of myopia may be exacerbated by laser [24]. One should also discuss the rare occurrence of anterior segment ischemia [25], cataract [22], pachyphakia [26], microcornea [26], angle-closure [27], vitreous hemorrhage [28], and progression of ROP with possible sequential retinal detachment [29] following laser therapy. If performing laser in the operating room, it is important to discuss the risks of general anesthesia on neurological development as well as mortality. The most likely “risk” of laser photocoagulation for type 1 ROP is that the disease continues to progress despite treatment and/or results in unfavorable structural outcomes. This may result from rapidly-progressive disease (there is a delayed onset of disease regression following laser, highlighting the paramount importance of timing in treating ROP), inadequate laser ablation with “skip” or untreated areas of the retina, and/or disease-modifying mutations affecting retinal vasculature including those in the WNT signaling pathway [30].

In our practice, infants are examined 1 week after laser and undergo continued surveillance for a total of 10 weeks to ensure complete and stable disease regression. If vascular engorgement persists for roughly 7 days following laser, this should be noted as significant and may indicate that further treatment is needed. If the disease fails to regress, re-activates, or progresses during the post-laser period of observation, one has the option to re-treat with laser (if skip areas identified), perform intravitreal anti-VEGF therapy, or perform vitreoretinal surgery (primarily done if a retinal detachment exists). Once treated, infants should be followed for life to evaluate for sequential retinal tear, detachment, or late disease recurrence.

The treating physician should have had dedicated and mentored training for treating ROP with laser, as the procedure is often challenging with many nuances and can be time-consuming to perform correctly. “Incomplete” laser may be a reason for the higher than previously reported laser failure rates noted in several studies exploring anti-VEGF therapies for ROP, as ophthalmologists today receive less dedicated laser experience than in the past [31].

## Treatment: anti-vascular endothelial growth factor (anti-VEGF) therapy for Type 1 ROP

One of the proteins produced during phase 2 is vascular endothelial growth factor (VEGF), a potent stimulator of angiogenesis. Angiogenesis plays a role in normal development and maintenance of the retinal vasculature. However, an overabundance of VEGF, in particular VEGF-A, can produce the pathogenesis that occurs in Type 1 Retinopathy of Prematurity [32]. Therefore, all therapies that are currently in use are designed to block the response of this protein leading to the development of abnormal blood vessels in the retina.

When ROP is classified as Type 1, one may choose to treat with laser therapy or “off-label” intravitreal anti-VEGF (anti-VEGF) therapy. Intravitreal *bevacizumab (IVB)* [33] (Avastin; Genentech, South San Francisco, CA), *ranibizumab (IVR)* [34] (Lucentis; Genentech, South San Francisco, CA), *aflibercept (IVA)* [35] (Eylea, Regeneron, Tarrytown, NY), and *conbercept* [36] (Chengdu Kanghong Biotech Co., Ltd., Sichuan, China) have been used in the treatment for type 1 ROP. Currently none of these drugs are FDA approved in the United States for the treatment of Type 1 ROP. However, ranibizumab has been approved for use in Type 1 ROP by the European Medicines Agency (EMA).

Detailed informed consent is critical when treating ROP with anti-VEGF therapies. It is important to discuss that anti-VEGF agents can enter the systemic circulation and potentially cause heart attack, stroke, pulmonary problems, bleeding, gastrointestinal problems, and mortality [37]. IVB (and to a lesser degree IVR) also reaches the systemic circulation and by extension the fellow eye in part due to its longer systemic half-life (20 days) compared to IVR (8 days) [38]. IVB has been noted to lower systemic VEGF levels for as long as 8 weeks after treatment [39], but the exact clinical significance of this is unknown. There have also been studies, mostly with IVB, demonstrating increased risk of neurodevelopmental delays in infants receiving anti-VEGF therapies [40], but ascribing the neurodevelopment delay to anti-VEGF therapy alone is challenging as the highest risk infants are also those most likely to be treated with anti-VEGF therapies. For example, another study comparing average Bayley-III scores for cognition, language, motor, and neurodevelopmental outcomes found no difference between infants who received off-label IVB with delayed laser therapy compared to those who received primary laser therapy [41]. Further prospective studies are needed to definitively assess the neurodevelopment risk of anti-VEGF therapy. While ROP and prematurity alone is associated with myopia, anti-VEGF therapy may be associated with less myopia than ablative laser therapy [42]. The general risks of intravitreal injection in an infant should also be discussed which includes retinal break, retinal detachment, ocular perforation, cataract, and infection (with possible loss of vision and/or the loss of the eye). Of utmost importance, anti-VEGF therapy for ROP is far from the “one and done” paradigm, as there is an extremely high likelihood (>90%) of needing additional examination under anesthesia, fluorescein angiography, and laser treatment in both eyes following anti-VEGF treatment. In addition, there are lifelong anatomical changes in the peripheral retina and increased risk for retinal detachment as a young adult (especially if the child was not also treated with laser therapy) which requires life-long monitoring [43].

Intravitreal bevacizumab (IVB) is a humanized monoclonal antibody (149 kDa) that blocks all VEGF isoforms and has been widely used “off-label” for the treatment of Type 1 ROP [44–46]. Compared to ablative laser therapy that results in a gradual decline of VEGF following treatment, IVB results in an acute drop in the concentration of intravitreal and systemic VEGF [39]. Lower dosages than this have been investigated [47], but lower dosages have been associated with a higher re-treatment burden. The first clinical trial employing anti-VEGF therapy “Bevacizumab Eliminates the Angiogenic Threat for Retinopathy of Prematurity” (BEAT-ROP) [46] compared IVB with laser in the treatment of Type 1 ROP. While BEAT-ROP did pave the way for future applications of anti-VEGF therapy in ROP, the study has several limitations including a higher than previously reported laser failure rate (42%), alteration of the primary study endpoint during the trial, a lack of long-term follow up, and the recommendation of intravitreal injection 2.5 mm posterior to the limbus (which poses a risk for retinal perforation compared to the 0.75 to 1 mm posterior to the limbus location recommended by the authors) [48]. The study title itself uses the phrase “eliminates the angiogenic threat for ROP,” which is not representative or practical. IVB (and other anti-VEGF agents discussed below) are revolutionary in the field of retina and ROP, but they serve primarily to “minimize threats” in the setting of proper surveillance rather than outright “eliminate threats” with a single injection. The current dose of IVB preferred by the authors’ is 0.625 mg of bevacizumab in 0.025 ml.

Intravitreal ranibizumab (IVR) is a humanized monoclonal antibody fragment (48 kDa) that has an affinity for all isoforms of VEGF [49]. However, when compared with IVB, IVR has a 5-20x fold greater potency on a molar basis, increased affinity for VEGF, and shorter serum half-life (2 h in adults). Results of the Phase III study “RAnibizumab Compared with Laser Therapy for the Treatment of INfants BOrn Prematurely With Retinopathy of Prematurity” (RAINBOW) indicate that intravitreal use of ranibizumab is an efficacious and well-tolerated treatment for ROP. In addition, no clear evidence of suppression of systemic VEGF levels was identified. In the RAINBOW trial, the need for re-treatment was similar between both of the drug groups and the laser group. In the laser group, 22 out of 52 infants (30%) required one or more additional treatments (including laser re-treatment). In the 0.1 mg ranibizumab group, 24 out of 55 infants (31%) had one more additional treatments, and in the 0.2 mg ranibizumab group, 23 out of 49 infants (31%) had one or more additional treatments. The long-term evaluation on safety data and functional outcomes (to 5 years of age) will be included in the ongoing RAINBOW extension study. In our clinical practice and collective experience with the 0.2 mg dose in over 300 injections, the recurrence rate is minimal and early and constant surveillance is critical. Success rates are more a reflection of the overall ROP care rather than the drug itself, as anticipating treatment, treating “on-time” at the first definite evidence of Type 1 ROP, close monitoring, timely re-treatment when indicated, and ongoing care are all critical components of a successful ROP treatment paradigm. The current dose of IVR preferred by the authors is 0.2 mg in 0.03 ml.

Intravitreal aflibercept (IVA) is a fusion protein (115 kDa) that binds multiple isoforms of human VEGF-A, VEGF-B, and placental growth factor [50]. IVA has been used for ROP in numerous reports [35, 51, 52], and is currently being compared to primary laser therapy via a study (NCT04101721) through Regeneron (BUTTERFLEYE).

SAFER is an acronym used to describe the intravitreal injection protocol in neonates [53], which consists of the following: Short needle, Antiseptic/antibiotic, Follow-up, Extra attention to detail, and Recheck every 1-2 weeks post-injection until complete retinal vascularization or additional laser has been administered to avascular retina. The **short** needle is a 32-gauge thin-walled stainless steel hypodermic needle 4 mm in length (TSK STERiJECT, Japan) [53]. The **antiseptic/antibiotic** is topical 5% or 10% betadine. This should be instilled before and after the injection. **Extra attention to detail** includes using the ora nomogram to determine the safest injection distance from the limbus in each quadrant [54], clean instruments, gloves, and masks for all involved in the injection procedure including nurses or respiratory therapists holding the infant. One should assess other risk factors for endophthalmitis including checking for the presence of nasolacrimal duct occlusion and conjunctivitis. Additionally, CPAP ventilation can transfer contaminated air from the nasopharynx to the injection field. The authors often find that long nasal prongs can easily and safely be inserted by the NICU team for the short procedure as an alternative to CPAP ventilation. Furthermore, unsheathed needles should not be held near the infant’s CPAP, nose, or mouth while maneuvering into position for the injection.

The injection is performed at bedside in the NICU on awake infants. Topical anesthetic is instilled into the eye followed by placement of an eyelid speculum. The use of additional sedation or anesthesia is per the discretion of the neonatologist but has not been found to affect adverse outcomes [55]. 5–10% Betadine drops are then instilled. Calipers are used to measure and mark the location for the injection 0.75 to 1.0 mm posterior to the temporal limbus using the ora nomogram for neonatal eyes [54]. The medication is injected using the 4 mm 32-gauge needle on a 1.0 cc syringe. If a 30 g ½ inch needle is used, care should be taken to insert the needle only ~1/3 of its length into the eye, as “hubbing” this needle can lead to retinal detachment and ocular perforation. The needle tip is kept parallel with the visual pupillary axis during the injection to avoid the infant lens which takes up more relative volume in the infant eye. Another drop of 5–10% Betadine is instilled, the retina and optic nerve are examined to ensure adequate perfusion, and the eyelid speculum is removed.

We then **Recheck** the patient 24–72 h post-injection to rule out endophthalmitis as well as every 1-2 weeks post-injection for disease reactivation, which in one study occurred in ~17% of eyes treated with IVB [33]. ROP re-activation has been noted to occur into teenage years in premature infants who did not reach pre-threshold treatment criteria for ROP and up to 69 weeks PMA [56] in those treated with anti-VEGF; thus close observation of these patients remains critical. Severe disease re-activation can result in fibrovascular membrane (re)proliferation with progressive tractional and effusive retinal detachment [57, 58].

## The role of fluorescein angiography and “Delayed” laser

**Fig. 1:**
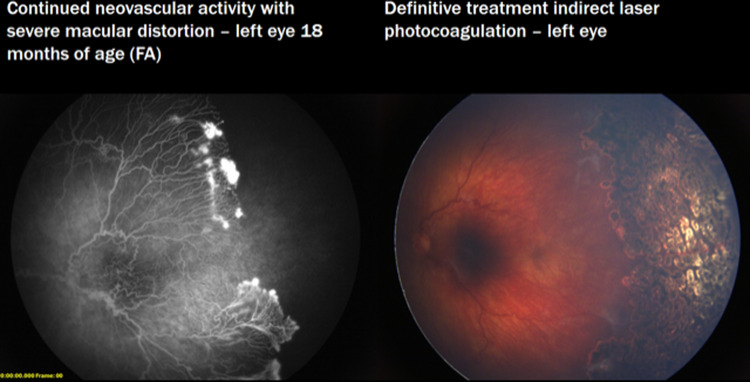
Delayed retinal vascular maturation following anti-VEGF therapy requires surveillance. Left eye of a child with type 1 ROP who had undergone two anti-VEGF injections at an outside hospital as an infant and did not receive follow-up with fluorescein angiography and laser until 18 months of age, disclosing continued neovascular activity with severe macular distortion. The fellow (right) eye of the child had also received three anti-VEGF injections at an outside hospital and was phthisical.

For all infants treated with anti-VEGF therapies who have not fully vascularized and for those with persistent mild or Type 2 ROP, we recommend performing fluorescein angiography (FA) by roughly 60 weeks PMA. This is important because the overwhelming majority of patients treated with anti-VEGF therapies will not fully vascularize (Fig. 1). The literature varies in describing this phenomenon, with one study finding that only 50% of the vasculature reached Zone III following IVR [59], and another study finding that only 3.3% of eyes following IVB fully vascularized (within 2 optic disc diameters of the termination of the retina) [60]. Persistent avascular retina increases the risk of ROP disease re-activation as well as retinal tears and detachments later in life [43, 61, 62]. In a large retrospective review of 363 eyes with untreated (mild or regressed) retinopathy of prematurity, 30.8% of eyes developed a retinal detachment [43]. While still a topic of debate, the authors believe that FA followed by laser may decrease these risks. When performing ‘delayed’ laser in an older infant, the persistence of tunica vasculosa lentis is less and therefore red or green laser may be sufficient. Sixty (60) weeks PMA was chosen as the time to perform FA with possible laser under general anesthesia for 4 primary reasons: (1) At 60 weeks PMA, the lungs and circulatory system in a neonate have matured substantially thereby decreasing the risks of general anesthesia; (2) At 60 weeks PMA, the retinal vasculature has usually advanced from its location when initially injected, but is unlikely at this point to continue substantial growth; (3) At 60 weeks PMA, the anesthesia guidelines of hospital systems may allow these children to be discharged from the hospital on the same day and obviate the need for overnight hospital admission; (4) At 60 weeks PMA, the examination in the clinic of such a large child is very difficult and often the zone of vasculature is not well visualized without FA.

## ROP modifying genetic mutations in the Wnt signaling pathway

The Wnt signaling pathway guides tissue differentiation in the developing fetus and plays several roles in adults including angiogenesis and maintenance of the blood-brain barrier (BBB) [63]. Patients with Wnt mutations involving (1) LRP5 (may be also associated with low bone mineral density), (2) NDP, (3) FZD4, and (4) TSPAN12 may develop a variety of retinal vascular diseases including Familial Exudative Vitreoretinopathy (FEVR), Norrie Disease (NDP mutation), and others [64]. It is becoming understood that a mother carrying a WNT signaling pathway mutation may have abnormal placental vascular development [65] that may result in placental insufficiency and possibly a premature infant. For example, Drenser et al. found that a FZD4 variant was associated with lower than normal birth weights for gestational age in infants with ROP compared with other premature infants [30] (present in 7.5% of patients with treatment requiring ROP compared to 1.8% in the 1000 genomes project). This type of premature infant may go on to develop “ROP,” but if carrying the same Wnt signaling mutation as his mother, may display features of “ROP” that are atypical including disease more severe and earlier than expected from their birth weight and gestational age, progressive disease despite timely and appropriate treatment, and/or ongoing disease activity following a period of quiescence. This unique combination of genetic mutations involving the Wnt signaling pathway, prematurity, and retinal vascular disease is termed “ROPER [66]” or “FROP” to denote the phenotypic overlap between ROP and FEVR in these infants and may be best diagnosed with fluorescein angiography [48]. While the long-term implications of this phenomenon are unknown, the diagnosis impacts treatment, follow-up (which may be longer than indicated by current screening guidelines and involve fluorescein angiography), and family planning. The possibility of a WNT signaling mutation also underscores the importance of performing fluorescein angiography in children treated with anti-VEGF therapy in order the reduce the possibility of disease recurrence and complications later in life.

## Conclusion

The prevention and treatment of type 1 ROP have improved dramatically over the past several decades with most centers reporting an average treatment rate of between 3–6% of all neonates screened. While treatment success may be somewhat influenced by the therapeutic modalities of choice, success is primarily dependent on the timing of intervention, close monitoring, timely re-treatment when indicated, follow-up past NICU discharge, and in cases with persistent ROP and prior anti-VEGF therapy, strong consideration for fluorescein angiography and laser therapy even in the absence of ongoing Type 1 ROP. In addition, the increasing availability of genetic testing may offer guidance in the surveillance and treatment of ROP, and telemedicine may allow for improved management of ROP [67].

The main challenge for the future in developed countries is the exponential growth of the number of micropremature infants defined as those with a gestational age of 24 weeks or less or a birth weight of less than 750 grams [68]. These infants may not conform to parameters advocated by ETROP and may require a different style of prophylactic monitoring in order to obviate severe retinal disease secondary to ROP. Some of the clues that we are often accustomed to such as classic “ plus” disease or stage 2–3 may not be the best harbinger of sight threatening disease in this micropopulation.

In developing countries, one of the primary issues and now the leading cause of childhood blindness is the “3rd epidemic” of ROP [69] secondary to an increase in the population of premature surviving neonates, poor nutrition, and inadequate oxygen control in some cases due to lack of oxygen blenders or complete lack of wall air or oxygen. Access to care and technologies need to be developed to address these issues [70]. Organizations including SIBA (iposc.org; Stop Infant Blindness in Africa) and Small World Vision (smallworldvision.org) are working diligently to resolve these inequalities.

Finally, the value of 80 years of vision cannot be underestimated on a personal, social, and economic basis for individuals and society. Neonatologists and ophthalmologists trained in the monitoring and treatment of ROP need to continue to work as a team to provide the best possible outcomes in our most tiny humans.
